# A Spirit Whisper

**Published:** 1892-08

**Authors:** 


### A SPIRIT WHISPER.

The following was sent from St. Paul as a special despatch, dated June 28, to the *Chicago Tribune*, in which it appeared June 29 :

Mrs. Cornelia Thomas, a handsome dressmaker of twenty-eight, living at No. 609 Decatur street, brought suit for divorce early in June against her husband, Eugene Thomas, alleging cruelty and inhuman treatment. Her sister, Mrs. Mary D. Phillips, of Seattle, Wash., knew a good deal about the treatment received from the husband, and so Cornelia sat down and wrote her all about the step she had resolved to take and asked her if she would not come to St. Paul and testify. Mrs. Phillips had just completed the sale of some Seattle real estate, so after putting sufficient money in her purse to meet her ordinary wants while absent she put $2,400 of the money from the sale of the real estate in the lining of her dress and sewed it in securely, thus to be provided in case of emergency.

The case came up June 22. Mrs. Phillips gave most satisfactory evidence for Cornelia and Cornelia secured her divorce. They spent a few days more together and Mrs. Phillips started home.

She was sleeping soundly in her berth when the train reached Tacoma. Then, as the train began to slacken its speed approaching the station, she was disturbed a little, and while in that frame of mind dreamed that she saw Cornelia take $1,000 of the $2,400 from the lining of her dress.

The surprise she experienced awakened her. What could such a dream as that mean? No, she would not allow herself to think for a moment that it could be true; and at that she placed her thoughts on things at home and how she should find them. But, try as she would to drown it, the horrible dream remained uppermost in her mind. There was one way to settle it, and she would just look and see if the money was there.

Of course it was, she thought; but when she got a look at it she would believe her eyes, and that would be the end of the dream. For a moment she shuddered at the thought of doubting her sister, but she arose in her berth and began searching for the lining of her dress. She had sewed the money in with red silk, and now it was sewed in with black silk. Hastily she ripped the seam open, and $1,000 of the money was gone.

Mrs. Phillips stepped off the train at Seattle and took the next train back to St. Paul. She arrived Monday and went at once to the office of County Attorney O'Brien. O'Brien procured a search warrant from the Municipal Court, also one for the arrest of Cornelia. The papers were placed in the hands of Lieutenant Murphy, and yesterday morning the Lieutenant, in company with Detective Daly and Mrs. Phillips, proceeded to the residence of Mrs. Thomas. Murphy read the search warrant to Cornelia and asked her to hand over the $1,000. She denied the charge emphatically; a search was instituted and a portion of the money found. She will be given a hearing Thursday.—*Chicago Tribune.*

---

**To Remove Pimples from the Face.**—Dissolve common salt in the juice of lemons, and with a linen cloth apply it to the parts affected.

